# The Red Line along the Gums

**Published:** 1890-03

**Authors:** 


### The Red Line along the Gums.

Dr. Snader says: Taking into consideration all the facts, I am forced to the conclusions:

That while the original observer of the red line in phthisical patients was correct in observation, he was incorrect in his deductions therefrom. The line itself is explicable on other and more reasonable grounds. A simple coincidence was mistaken for an associated condition.

That the line is not a diagnostic sign of phthisis at all, but of a disease of the gums.

That, unfortunately, one can not diagnose a case of phthisis by an examination of the gums.

That aside from tubercularization, lead poisoning, and scurvy, a changed gum line, in the present state of our knowledge, is not diagnostic of phthisis, nor of any other systemic disease.

That as a disease of the gums, the red line may be a local disease from neglect of the teeth, which may find a genuine predisposition in general connective-tissue relaxation.

That the red line along the gums, which can probably be found in any disease, gives rise to sufficient debility to cause a loss of general tissue tone, if sustained long enough to allow of a deposition of dental *debris* between the gum edges and the teeth.

That in cases of hæmoptysis, where neither cardiac nor pulmonary lesions are discoverable by physical exploration, the gums should be examined.

That the exact value of the red line along the gums as a diagnostic sign of phthisis is naught.

That the red line is significant of disease of the gums, due to improper care of the teeth, excessive accumulation of tartar, or to general systemic tone-relaxation, of which the red line is simply a local manifestation.—*Hahnemannian Monthly.*

---

Some one has discovered that a week galvanic current, which will sometimes cure a toothache, may be generated by placing a silver coin on one side of the gum and a piece of zinc on the other. Rinsing the mouth with acidulated water is said to increase the effect.—*Weekly Med. Review.*

